# 

**Published:** 1910-11-12

**Authors:** 


CONTENTS.

| | PAGE |
|---|---|
| **A Bad Beginning** ... | 185 |
| **Winter Fads** ... | 186 |
| **Annotations—** | |
| Postmen and Lifts — Jews in Medicine — Prosecutions in Paddington ... | 187 |
| **Medical Opinion and Movement** ... | 188 |
| **Hospital Clinics—** | |
| Some Cases of Diabetes. By Nestor Tirard, M.D., F.R.C.P.... | 191 |
| **Special Article—** | |
| The Tuberculin Test of Cattle as a Guarantee of Tubercle Free Milk ... | 193 |
| **Medicine—** | |
| The Detection of Blood Pigments in the Fæces ... | 194 |
| **Diseases of Children—** | |
| Acute Primary Pyelitis—Coli Bacilluria—in Children ... | 195 |
| **Motoring Notes -** | |
| Some Points in the Choice of a Car ... | 197 |
| **Parliament and Publications—** | |
| The Dublin Hospitals—The Health of the Metropolitan Police—The Reassembling of Parliament—Railway Accidents—Health in Reformatory and Industrial Schools ... | 198 |
| **News and Events of the Week** ... | 199 |
| **Appointments and Resignations** ... | 200 |
| **Obituary** ... | 200 |
| **Reports on the Hospitals of the United Kingdom—** | |
| LV.—Essex County Hospital, Colchester ... | 201 |
| **Hope Hospital, Salford** ... | 203 |
| **The Becket Hospital, Barnsley** ... | 204 |
| **The Week's Work—** | |
| The Jury "Do not Appreciate" — Hospitals and Expert Assistance—This Week's Drug Market ... | 205 |
| **The Hospital World** ... | 206 |
| **Special Institutional Articles—** | |
| The After-Care and Employment of Consumptives ... | 207 |
| **Institutional Notes and News** ... | 210 |
| **The Question Box—** | |
| I.—Ambulance Arrangements for Hospitals ... | 211 |
| II.—Private Rooms in Fever Hospitals ... | 213 |
| **Gifts and Bequests** ... | 214 |
| **Jottings** ... | 214 |

